# FEA-Swin: Foreground Enhancement Attention Swin Transformer Network for Accurate UAV-Based Dense Object Detection

**DOI:** 10.3390/s22186993

**Published:** 2022-09-15

**Authors:** Wenyu Xu, Chaofan Zhang, Qi Wang, Pangda Dai

**Affiliations:** 1 Hefei Institutes of Physical Science, Chinese Academy of Sciences, Hefei 230031, China; 2 Science Island Branch of Graduate School, University of Science and Technology of China, Hefei 230026, China

**Keywords:** object detection, aerial images, transformer-based, foreground enhancement attention, improved bidirectional feature pyramid network

## Abstract

UAV-based object detection has recently attracted a lot of attention due to its diverse applications. Most of the existing convolution neural network based object detection models can perform well in common object detection cases. However, due to the fact that objects in UAV images are spatially distributed in a very dense manner, these methods have limited performance for UAV-based object detection. In this paper, we propose a novel transformer-based object detection model to improve the accuracy of object detection in UAV images. To detect dense objects competently, an advanced foreground enhancement attention Swin Transformer (FEA-Swin) framework is designed by integrating context information into the original backbone of a Swin Transformer. Moreover, to avoid the loss of information of small objects, an improved weighted bidirectional feature pyramid network (BiFPN) is presented by designing the skip connection operation. The proposed method aggregates feature maps from four stages and keeps abundant information of small objects. Specifically, to balance the detection accuracy and efficiency, we introduce an efficient neck of the BiFPN network by removing a redundant network layer. Experimental results on both public datasets and a self-made dataset demonstrate the performance of our method compared to the state-of-the-art methods in terms of detection accuracy.

## 1. Introduction

Object detection is the process of localizing and classifying objects in an image. It is an essential task in the computer vision field and attracts much interest from both the academic and industrial communities. Inspired by the success of deep learning (DL), the performance of object detection has been dramatically improved. With the development of various technologies, unmanned aerial vehicle (UAV) platforms are widely used in the remote sensing field because of their attractive proprieties, including high flexibility, ecological benefits, and so on.

Object detection plays a pivotal role in many UAV applications, such as hazard monitoring [1], intelligent agriculture [2], traffic management [3], security and protection [4], etc. Although most existing object detection approaches demonstrate significant performance in ground-based images, they show limited performance in UAV aerial images, as UAV aerial images are completely different from ordinary images and characterized by complex backgrounds and small and dense objects. Thus, further improving the performance of object detection for UAV is attracting much attention and has become a hot area of research.

For a prolonged period, convolutional neural networks (CNNs) have been the dominant player in object detection. Numerous impressive object detection approaches have been proposed, such as R-CNN [5], Faster R-CNN [6], Cascade R-CNN [7], YOLO [8], and SSD [9]. These approaches achieve remarkable performance in natural scene images [10], whereas aerial images pose challenges to these approaches. The performance of CNN-based object detection approaches will degenerate or even collapse, because UAV aerial images are characterized by complex backgrounds and small and dense objects. For dense target detection in aerial images, the negative effect is very apparent due to the inflexible receptive field of convolutional kernels. Many efforts have been implemented to improve the performance of object detection. One common solution is to obtain a density map by first using density generation networks, then cropping the image blocks according to the density map, and finally detecting the targets and fusing the detection results of the density blocks and the whole image [11,12,13,14]. The drawback of this solution is that the additional network significantly adds to the model’s computational volume. Another effective solution is to use the attention mechanism. CBAM [15], MSCA [16], and other attention models [17,18,19] have emerged to boost the performance of object detection by exploiting positional information through reducing the channel dimension of the input tensor with large-size convolutional kernels. However, most of these approaches embed the attention mechanism into deep convolutional networks. They strengthen the contextual connection to some degree but fail to model the long-range dependencies, which are critical for dense object detection in aerial images.

Recently, the Transformer network provides qualitative performance in the field of computer vision. It is worth mentioning that the Vision Transformer (ViT) [20] network demonstrates that a pure attention-based model leads to better results than a CNN-based model. Swin Transformer [21] is the most representative and impressive structure. It models the local relationship only at each stage while continuously reducing the feature map width and height and expanding the receptive field. Thus, it can be employed as a universal backbone for the main downstream visual tasks. Recent studies have shown transformer-based object detection approaches have shown significant performance [22,23,24]. These methods have scored extremely well in large natural scene datasets such as MS COCO [25] and ImageNet [26]. Many attempts have also been carried out with transformer-based models for target detection in remotely sensed images and aerial images [27,28,29]. However, the accuracy of existing transformer-based object detection approaches is still insufficient in challenging conditions. We mainly discuss it from two sides.

First, by revisiting the object detectors for aerial images, we uncovered a potential problem: extracting the correlation information between contexts is urgently needed [30,31,32,33,34]. From this point of view, Swin Transformer does not cope well with the detection of dense objects when responding to aerial images due to its failure to notice the connection between neighboring targets [35]. Within the framework of CNNs, there are two endeavors presenting the idea of foreground enhancement that are very worthwhile. Zheng et al. designed FarSeg [30], which consists of two modules: a foreground scene relationship module and foreground perception optimization. The former reduces false positives by learning symbiotic relationships between scenes and foregrounds to associate foreground-related contexts with enhancing foreground features. The latter mitigates the foreground-context imbalance problem by suppressing multiple simple examples in the background and focusing the model on the foreground. Song et al. argue that enhancing object-related features can help reduce false and missed detections in aerial images [36]. Therefore, they create a new branch called the foreground enhancement module (FEM) after the feature pyramid network by first regressing the masks that represent the foreground and background. Next, the obtained mask enhances the original fused features, and the detector’s ability to distinguish between foreground and background can be improved. However, current transformer-based models lack such work. Motivated by the endeavors noted above, we have an intuitive and novel idea to add the foreground enhancement mechanism into the backbone of the Swin Transformer in the form of attention operation, known as the foreground enhancement attention block (FEAB).

Second, the neck is the key element in the target detection framework that carries on from top to bottom. It reprocesses and rationally utilizes the important features extracted from the backbone to facilitate the next step of the head for concrete task learning. The most common current use of the neck is BiFPN [37]. Low-level features with high resolution but weaker semantics are downsampled and combined with high-level features to create feature representations with both high resolution and strong semantics. However, the operation of successive downsampling causes the feature map to drop a considerable amount of fine-grained information [38,39]. This is detrimental to the detection of small objects. To tackle this problem, we have designed an improved BiFPN that incorporates operations of skip connection and strike out of redundant output nodes.

To summarize, the contribution of this article is fourfold.

1. To the extent we are aware, we are the first to introduce a foreground enhancement attention block (FEAB) in the original Swin-tiny backbone to bring more contextual information and learn more recognizable features and investigate its effectiveness in aerial image object detection tasks. Moreover, the FEAB module we introduced in the backbone can theoretically be inserted into other existing hierarchical vision transformers.
2. We additionally propose a straightforward and efficient weighted bi-directional feature pyramid network (BiFPN) for efficiently fusing feature maps with context information from different stages of the encoder.
3. We have created a self-collected dataset around the lab, which currently has 2000 images, targeting both pedestrians and vehicles, which we have annotated and made public. The download link is given at the end of this article.
4. Finally, we provide an in-depth analysis of the impact of each of the two critical components in FEA-Swin on detection accuracy. Our proposed method achieves competitive performance metrics on the VisDrone, NWPU VHR-10, and our self-collected dataset, exceeding the best currently available universal models.

## 2. Related Work

Aiming to better understand and design aerial image target detection models, this section presents relevant work, including Vision Transformer, Swin Transformer, attention mechanism, and object detection in aerial images.

### 2.1. Object Detection in Aerial Images

Object detection is one of the fundamental problems in the field of computer vision. For a long time, due to many difficulties such as slow detection speed, insufficient generalization ability, and complicated manual feature design of traditional methods, target detection has not been widely implemented in practical scenes. In recent years, with the rapid development of artificial intelligence, emerging technologies represented by deep learning have made breakthroughs in computer vision, natural language processing, speech recognition, and other research fields by virtue of their excellent generalization ability. At the same time, driven by the wave of technology, UAVs have the advantages of long endurance, low power consumption, and real-time processing and transmission, which enriches the application of target detection on UAVs and makes them gradually become the focus of attention.

UAV aerial images are characterized by complex backgrounds and small and dense targets due to the imaging perspective being different from natural scene images, which leads to even more challenges for target detection in aerial images [40]. Due to the small target size and dense distribution of targets, it is difficult to achieve satisfactory results by directly applying a generic target detector to aerial images. To cope with this problem, the following methods are proposed.

- ClusDet [11] proposes an end-to-end aerial target detection framework that combines target clustering and detection. It consists of three main basic components: a cluster proposal network (CPNet), which is used for target clustering to generate target cluster regions; a scale estimation network (ScaleNet), which estimates the scale of target clusters; and a dedicated detection network (DetecNet), which performs target detection on the cluster regions normalized to each scale.
- DMNet [12] leverages density maps to do target detection in aerial images. Density maps come from a similar field, crowd counting. Density maps can reflect the distribution of targets in an image. In crowd counting, the targets are highly dense and unevenly distributed, and the scale of individual targets is small, which is highly similar to the target distribution in aerial remote sensing datasets. The proposed DMNet consists of three main steps: (1) density map generation network; (2) segmentation of the input map into foregrounds based on the density map; (3) target detection using the generated foregrounds.
- GLSAN [13] proposes an end-to-end global–local adaptive network. It consists of three main components: a global-local detection network (GLDN), an adaptive region selection algorithm (SARSA), and a local super-resolution network (LSRN). The method integrates a global–local fusion strategy into a progressively scale-varying network to perform more accurate detection.
- UCGNet [14] proposes a network based on unsupervised clustering guidance. First, a local location module (LLM) is proposed to predict binary images using an attention mechanism. The binary map can represent the location of the target presence in the image. Second, an unsupervised clustering module (UCM) is proposed to cluster these points into some clusters (clusters). To enhance the effectiveness of these clusters, the authors sample 1000 points from all pixels covering the target using the farthest point sampling strategy. Each cluster corresponds to a region. Third, these sub-regions are cropped down. Finally, the global fusion module (GMM) is used to join all candidate frames to obtain the final detection results.

### 2.2. Attentional Mechanisms

In daily life, human eyesight quickly scans the global environment to obtain the target area to be focused on, which is generally known as the focus of attention, and then devotes more attentional resources to this area to obtain more detailed information about the target, while suppressing other useless information. Motivated by that phenomenon, attentional mechanisms were introduced to computer vision with great success.

The attention mechanism is capable of focusing high weights on important information and low weights to ignore useless information, with the ability to dynamically adjust the weights, making the model more responsive to different situations.

Attention mechanisms in computer vision are divided into six types, including the basic four: (1) temporal attention, (2) branch attention, (3) spatial attention, and (4) channel attention, versus a mixture of two: (5) combined temporal attention and spatial attention and (6) spatial attention and channel attention. The most commonly used of these are (3), (4), and (6). Several representative efforts are presented below.

- SENet [17]: It pioneered channel attention. At the heart of SENet is a squeeze and excitation (SE) block that models the global picture, observes phase relationships between different channels, and improves the learning capability of the model. The disadvantage of this is that the global average pooling in the squeeze module cannot handle complex features. The fully connected layer in the excitation module also adds redundant operations.
- Non-Local [18]: It uses a spatial attention mechanism to directly model any two locations in the image, capturing long-range dependencies. The set of locations can be spatial, temporal, or spatio-temporal. It has the advantage that it can be fused with other operations for insertion into other networks, but, again, requires a larger amount of operations.
- RAN [19]: A residual attention network (RAN) is proposed, which stacks multiple attention modules. Its advantages are that it can capture mixed attention and is a scalable convolutional neural network. However, the proposed bottom-up structure fails to fully utilize the global spatial information. In addition, direct prediction of a 3D attentional map has a high computational cost.

### 2.3. Vision Transformer

ViT is the first visual detector that uses a pure transformer framework. ViT divides the input image into multiple patches (16 × 16), and then projects each patch into a fixed-length vector to feed the Transformer. The subsequent encoder operation is exactly the same as in the original Transformer. As the images are classified, a special token is added to the input sequence, and the output corresponding to this token is the final category projection.

ViT proves that deep models built with pure self-attention blocks can also perform outstanding results in various vision tasks. However, ViT also has obvious limitations, such as huge data requirements, a limited number of stacked layers, and the inability to encode locations in the model itself.

### 2.4. Swin Transformer

Regarding the application of Transformer from natural language processing to the computer vision field, the main adjustment is the scale of the visual image and the high-definition issue. Despite the huge sensation ViT has caused in the computer vision community, it is not perfect and still has some drawbacks. One of the most serious of these is the compatibility of ViT with high-resolution images, because its computation generates a quadratic complexity associated with the image size, leading to a high number of tokens and a very high computational effort for self-attention requirements.

**Figure 1:**
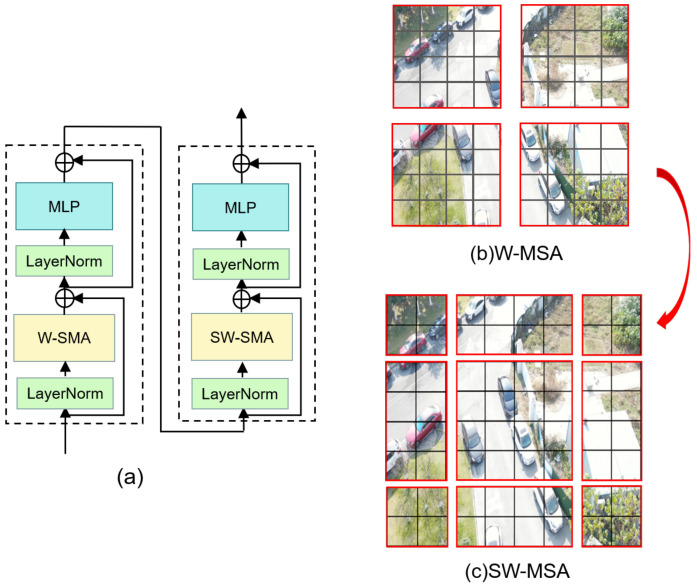
(**a**) Two consecutive Swin Transformer blocks; (**b**,**c**) is the description of the shift window method for calculating self-attention in the Swin Transformer framework.

In this case, Swin Transformer introduces two key concepts to solve the problems faced by the original ViT, which are hierarchical feature mapping and windowed attention transformation. The model designs sliding windows to compute only the self-attention of all tokens inside that window to reduce the computational effort. At the same time, the hierarchical structure allows the model to have adaptive modeling with linear complexity for images of different scales. In fact, the name of Swin Transformer comes from “Shifted window Transformer,” and the most vital component of Swin Transformer is shown in Figure 1.

## 3. Methods

### 3.1. Overview of the Proposed Framework

**Figure 2:**
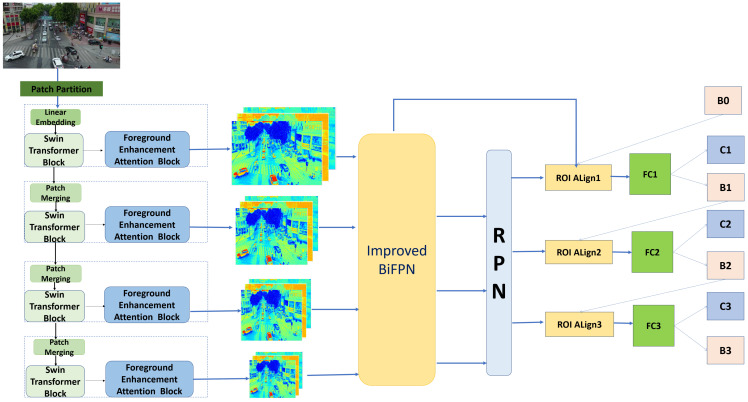
Architecture of FEA-Swin.

We now give an overview of FEA-Swin. First, our proposed FEA-Swin adopts the classical backbone–neck–head architecture, and the overall framework is shown in Figure 2. In the backbone, we designed and introduced FEAB as the backbone of our FEA-Swin, based on the primitive Swin-tiny. The backbone network here is described below as an encoder. Next, we designed an improved version based on the original BiFPN in the neck, introducing the skip connection and strike output node operations. Finally, we used the most popular head of Cascade R-CNN in the head.

### 3.2. FEAB in Backone of FEA-Swin

**Figure 3:**
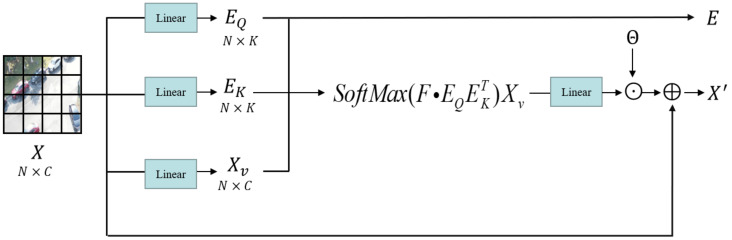
The detailed structure demonstration of FEAB.

**Figure 4:**
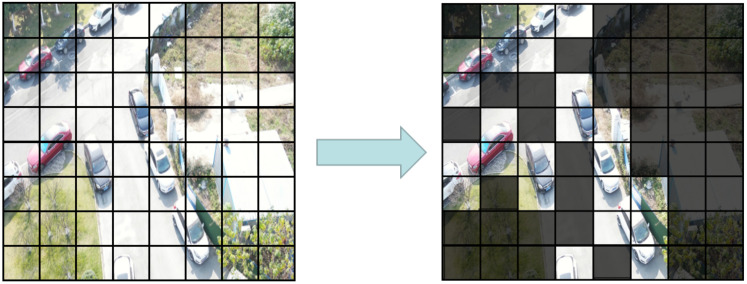
A visual schematic diagram of FEAB.

The RGB image of size $R^{H\times W\times 3}$ is partitioned into non-overlapping patches of size $R^{4\times 4}$. These patches are then used as tokens and sent as input to the encoder, for which we use Swin-tiny here. Before the first transformer layer, there is a linear embedding operation that converts the features to the required dimension (denoted as C). The encoder extracts features in four stratified stages. Each stage in the encoder is assembled by a transformer layer and a context layer in tandem, and the output of the transformer layer is fed into the context layer. The transformer layer is composed of $N_{S}$ standard Swin-tiny transformer blocks (Figure 1) stacked to extract features from the original image, whereas the context layer is composed of $N_{F}$ FEAB (Figure 3) stacked to enhance foreground contextual information, generating context-prior maps and updating the feature values with them. The detailed structure of FEAB and the diagram of the reasoning process (Figure 4) are shown below.

In each standard Swin-tiny transformer block, there is a shift window, or window. The attention mask set determines which attention is used to limit what can be seen at each location in the attention. The window attention is similar to the transformer block in ViT with the addition of relative position encoding. It computes attention only for tokens within a window, whereas shifted window attention computes attention for tokens in different windows belonging to non-overlapping regions. Both attention operations have linear time complexity, making the design better for high-definition image feature representation. To obtain a hierarchical feature map, patch merging is utilized at the beginning of each stage, starting from the second stage, to reduce the number of tokens. The size of the resolution of the feature map output at each of the four stages is $\{\frac{H}{4}\times \frac{W}{4}\times C$, $\frac{H}{8}\times \frac{W}{8}\times 2C$, $\frac{H}{16}\times \frac{W}{16}\times 4C$, $\frac{H}{32}\times \frac{W}{32}\times 8C\}$. Following the self-attention approach, we divide the input features X into Q, K, and V, which represent query, key, and value matrices, respectively. Similarly, we introduce a relative position bias $RPB\in R^{W\times W}$ in the calculation of self-attention:

(1) $$Attention\left(Q,K,V\right)=SoftMax\left(\frac{QK^{T}}{\sqrt{D^{k}}}+RPB\right)V,$$

where $D^{k}$ represents the dimension of key. After the last Swin Transformer block of the current layer, the output feature map is fed to the context layer as input.

High-quality context information proves to be an essential part of the target detection task. Therefore, the context layer that follows is the key component of the encoder and the most innovative part of this article. Each context layer consists of $N_{F}$ FEAB. The FEAB enhances the foreground features by weakening the background features to concentrate cross-window attention on the foreground region rather than the entire feature map. Following Swin’s approach, in order to limit the computational cost to a linear scale, we divide the input X′ of FEAB into three components, $E_{Q}$, $E_{K}$, and $E_{V}$, representing context query, context key, and context value, respectively. By multiplying the input features X′ with the randomly initialized learnable matrices $W_{Q}$, $W_{K}$, $X_{V}^{\prime }$, respectively, we linearly project the input features into the context space and obtain $E_{Q}$, $E_{K}$, and $X_{V}^{\prime }$. The first two expressions, $E_{Q}$ and $E_{K}$, are foreground-enhanced query and key, which are normalized by a softmax function and used to update the value of $X_{V}^{\prime }$. Moreover, we add a learnable scalar constant Θ after the linear layer for smooth fine-tuning. This is because firstly the scalar suppresses the scale of the converter during initialization. Second, the attention value of the transformer may decay as the weight decreases during training, and the learnable scalar we have added compensates exactly for this, keeping the output within an acceptable range. Our foreground enhancement attention equation is given below:

(2) $$FEAttention\left(E_{Q},E_{K},X_{V}^{\prime }\right)=SoftMax\left(F\cdot E_{Q}E_{K}^{T}\right)X_{V}^{\prime }+\Theta ,$$

where the foreground mask at the feature pixel location (x, y) is

(3) $$F\left(x,y\right)=\left\{\begin{array}{cc} 1, & if\;M(x,y)=1\\ 0, & if\;M(x,y)=0 \end{array}\right..$$

In this case, $M\left(x,y\right)\in {\left\{0,1\right\}}^{H\times W\times C}$ is a binarization function (with threshold 0.7) whose value is obtained by mask prediction from the (*x*, *y*) position of the original input feature *X*.

### 3.3. Improved BiFPN as the Neck of FEA-Swin

Objects in aerial drone images are usually small and dense, and there are limitations in the feature representation capability of a single layer of backbone. Thus, in an effort to efficiently and quickly fuse different scale feature maps from different layers of the encoder, we demand an advanced feature fusion network. In general, the output feature map $P^{O}=f\left(P_{i}^{I}\right)$, where $P_{i}^{I}$ stands for the feature map of the *i*th level and *f* stands for feature fusion method.

**Figure 5:**
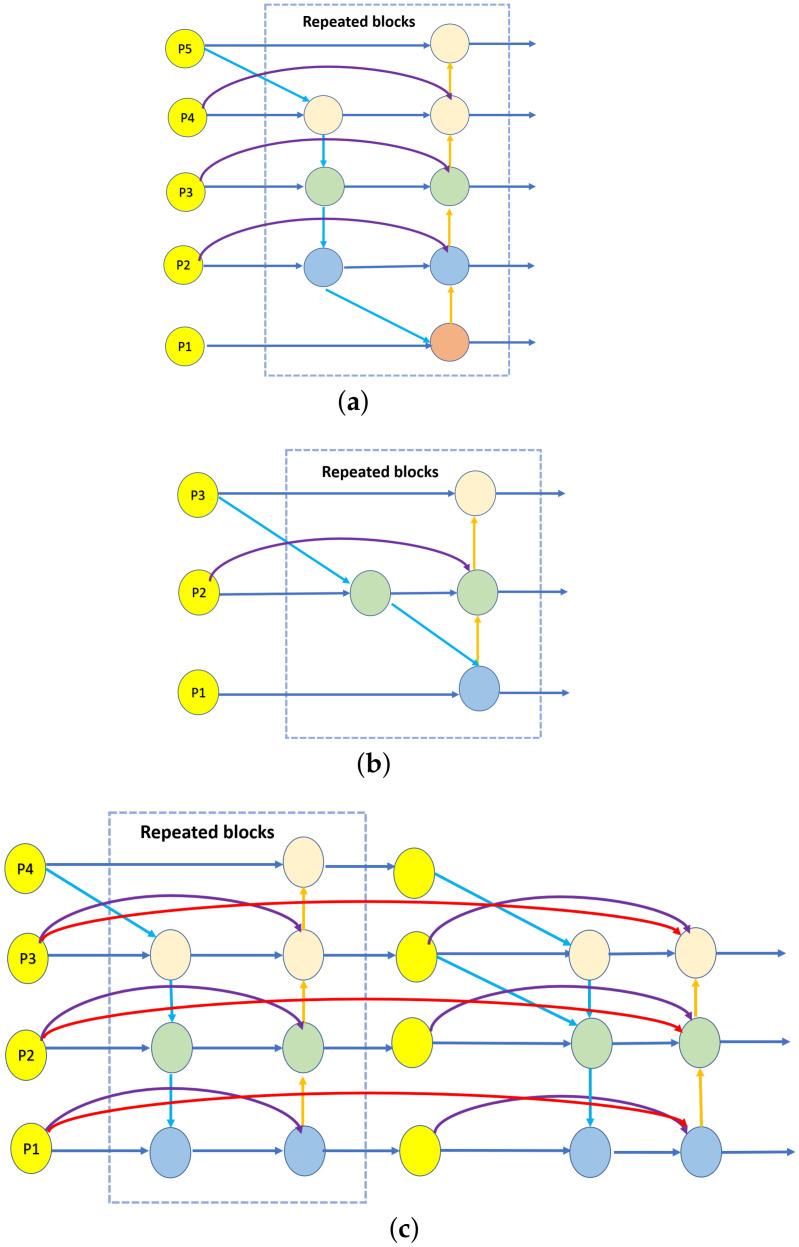
(**a**) Original BiFPN; (**b**) 3-level input and output BiFPN; (**c**) our improved BiFPN.

Currently, one of the state-of-the-art FPNs is the weighted bi-directional feature pyramid network (BiFPN), as shown in Figure 5a. There are two main contributions of BiFPN, that is, cross-scale connection and weighted feature fusion. Particularly, the former allows the aggregated feature graph to have more context information. The latter is proposed to allow the network to understand the contribution of each input feature to the output result. The algorithm for weighted feature fusion is described as follows:

(4) $$O=\sum_{i}\frac{\omega_{i}}{\epsilon +\sum_{j}\omega_{j}}\cdot I_{i},$$

where $\omega_{i}$ is a learnable weight and is greater than or equal to 0 by the ReLu function that follows immediately after; ϵ = 0.0001 is used to ensure numerical stability; $I_{i}$ represents the input feature map of the *i*th level.

According to the idea of BiFPN, when our work requires the input feature map to be four layers, then the structure shown in Figure 5b should be used. Unfortunately, this structure enables the output feature maps of some layers to be inaccessible to all layers after aggregation. Consequently, we implement an improved BiFPN. The structure of our improved BiFPN is shown in Figure 5c. The backbone delivers four levels of features at different scales that are treated as inputs; following the idea of BiFPN, the first n repeated blocks all have 4-level input and output. The last block of the original BiFPN has four levels of output, and each aggregated feature map appears as an input to the region proposal network (RPN). In this case, the output of each level straightforwardly influences the final result. However, our improved method only takes 3-level output in the last layer. In addition, we designed the skip connection operation to prevent the information loss of small objects. For example, we denote the *i*th level input feature map of the first layer as $P_{i}^{F}$, the feature map for the intermediate iterations as $P_{i}^{T}$, and the input and output feature maps are $P_{i}^{I}$ and $P_{i}^{O}$, respectively. The second level output feature map of the last layer is calculated as:

(5) $$P_{2}^{O}=Conv\left(\frac{\omega_{1}^{{}^{\prime }}\cdot P_{2}^{I}+\omega_{2}^{\prime }\cdot P_{2}^{T}+\omega_{3}^{\prime }\cdot Resize\left(P_{1}^{O}\right)+\omega_{4}^{\prime }\cdot P_{2}^{F}}{\omega_{1}^{{}^{\prime }}+\omega_{2}^{\prime }+\omega_{3}^{\prime }+\omega_{4}^{\prime }+\epsilon }\right)$$

## 4. Experimental Results and Discussion

In this section, to evaluate the performance of our method, we perform several experiments on both public datasets and one self-collected dataset. We compare the proposed FEA-Swin with the state-of-the-art object detection systems, including Swin Transformer-tiny with FPN [21], one-stage object detectors (RetinaNet [41], YOLOF [42]), and two-stage object detectors (Cascade R-CNN, Faster R-CNN). We implement the proposed FEA-Swin on the MMDetection2D [43] platform under the Ubuntu operating system. All experiments have been run on an Intel Core i9-9900K CPU at 3.6 GHz with 16 GB RAM and an NVIDIA GTX 2080Ti GPU. In addition, we adopt the deep learning framework of Pytorch with CUDA10.1 and cuDNN7.6.5 for computational acceleration.

### 4.1. Datasets

In this paper, the proposed system is tested on two publicly available datasets (Visdrone-2021-Det [44] and NWPU VHR-10 [45]) and a home-made dataset. The details of these datasets are as follows.

**Figure 6:**
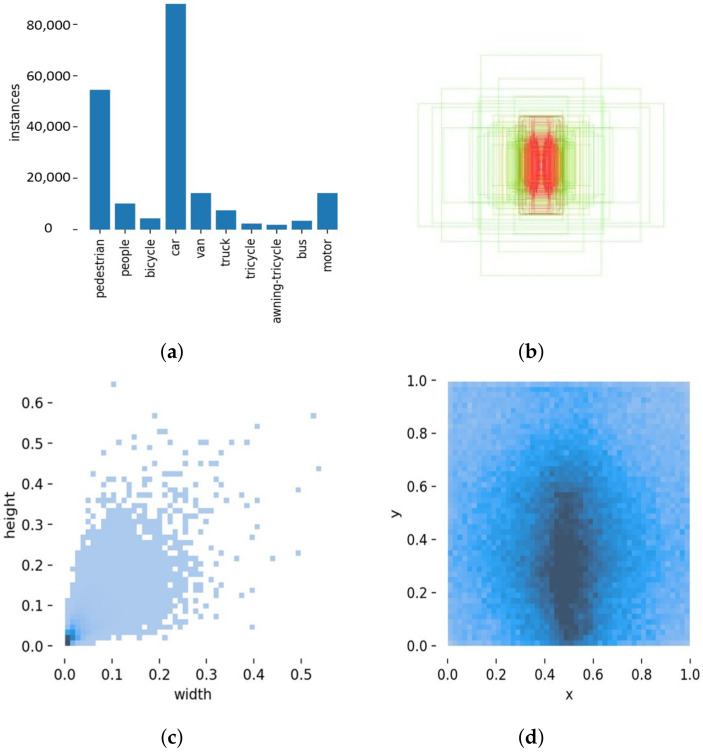
Details of the Visdrone dataset: (**a**) number of labels in various categories; (**b**) visualization plot of all labeled boxes aggregated at the center; (**c**) statistical plot of the width and height of all labels; (**d**) heat map of all labels occurring at the image positions.

**Visdrone** [44] is one of the most popular and challenging UAV aerial photography datasets. This dataset provides four sets of 10,209 images for 2D object detection tasks, including 6741 images in the train set, 548 images in the validation set, 1610 images in the test-dev set, and 1580 images in the test-challenge set. A total of 342,391 labels are manually annotated and classified into 10 categories. Because the official evaluation portal for the test-challenge set is no longer available, we used the test-dev set to evaluate our method. The details of the Visdrone dataset are presented in Figure 6. First, we enumerated the number of labels in each category, which shows that pedestrians and vehicles make up the majority. We then visualized all the annotation boxes and present them together in a subplot, which tells that there are more small targets. Next, we showed the distribution of the height (vertical) and width (horizontal) of each box, which reveals that most of the boxes are within (0.3 m, 0.3 m) of each other. Finally, we placed the centroid of each annotation box in a subplot to create a heat map of the target distribution locations, and we can see that the targets appear mostly in the middle, with the rest evenly distributed around them.

**Figure 7:**
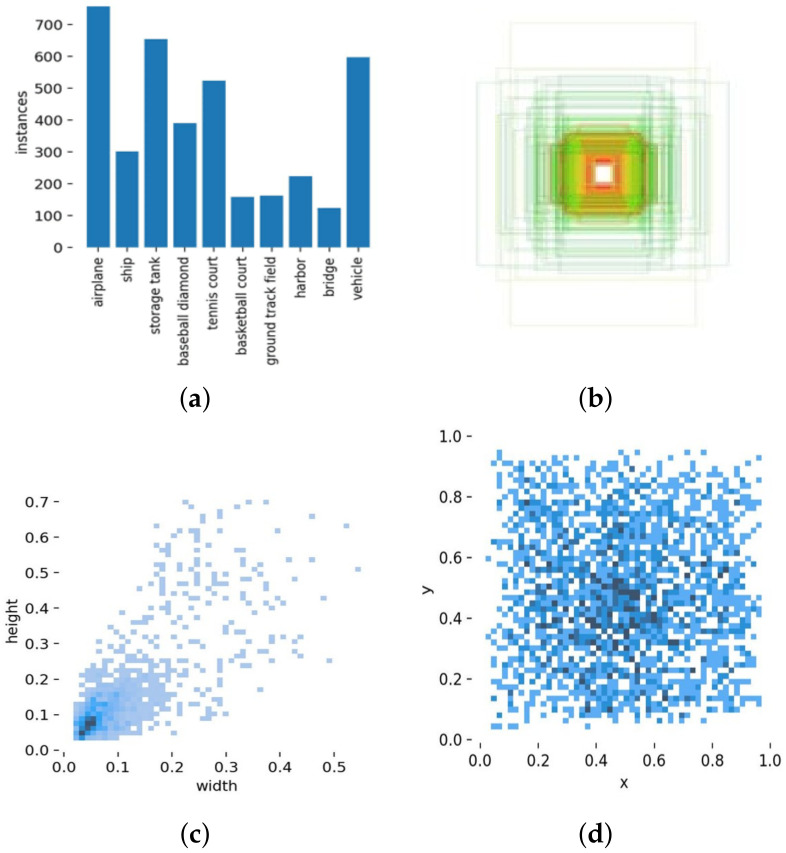
Details of the NWPU VHR-10 dataset: (**a**) number of labels in various categories; (**b**) visualization plot of all labeled boxes aggregated at the center; (**c**) statistical plot of the width and height of all labels; (**d**) heat map of all labels occurring at the image positions.

**NWPU VHR-10** [45] is a classic high-definition aerial object detection remote sensing dataset. Images in this dataset are selected and cropped from Google Earth and Vaihingen datasets, with a maximum resolution of 0.5 m and a minimum resolution of 2 m and contain 10 categories. These images vary in size, being roughly 500 pixels high by 1000 pixels wide, and are divided into sets of 650 positive images and 150 negative images; the negative ones do not provide any labeled targets. The details of NWPU VHR-10 are presented in Figure 7. First, we enumerated the number of labels in each category, which shows that the dataset is relatively homogeneous in terms of the number of categories. Then, we visualized all the label boxes and presented them together in a subplot, which indicates that the number of small targets is close to the number of large targets. Next, the distribution of the height (vertical) and width (horizontal) of each label box was displayed, and it can be seen that most of the label boxes are within (0.2 m, 0.2 m) of each other and that there are particularly many targets smaller than (0.1 m, 0.1 m). Finally, we placed the centroid of each annotated box in a subplot to produce a heat map of the target distribution locations, which reveals that the target occurrences are evenly distributed throughout the image.

**Figure 8:**
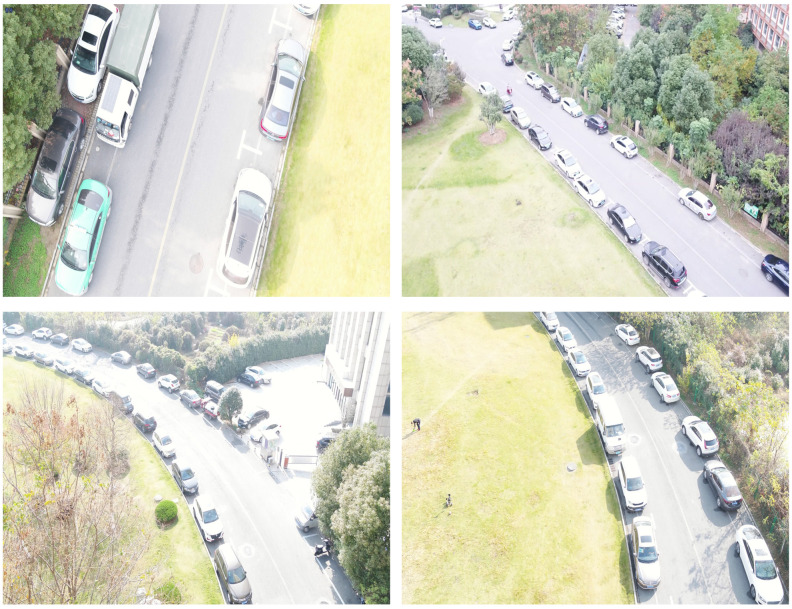
Some selected unprocessed images of our self-collected dataset.

**Self-collected dataset**: In addition to the above public datasets, we collected a dataset to further evaluate our method. We used a DJI drone to collect images under different challenging environments, including solid light changing, dense parked vehicles, etc., as shown in Figure 8. We used artificially controlled drone flights to photograph around our laboratory (Hefei, China). Our self-collected dataset provides two thousand images, and includes human and vehicle two categories. Our images are 1920 pixels wide and 1080 pixels high, where the ratio of (large target: medium target: small target) is approximately 2:5:3. A complete set of 12,500 labels was manually annotated with the LabelMe [46] software. In view of the time and labor consumption, we have used the most common rectangular marker bounding box. The dataset is now open access and the link is given at the end of this article.

In addition, we converted all labels to standard VOC2007 format. Furthermore, we observed the common practice of training on the training set, tuning the hyperparameters, checking the convergence with the validation set, and finally testing on the test set. It should be noted that we do not perform additional data augmentation on the images beyond the basic random flipping and cropping.

### 4.2. Implementation Details

**Figure 9:**
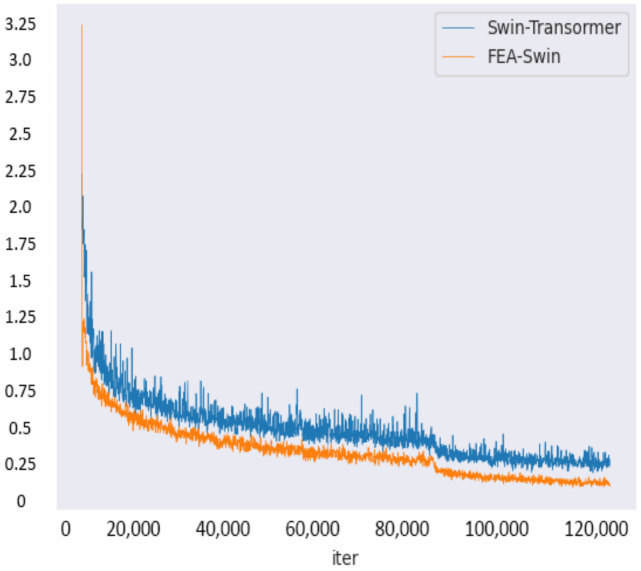
The loss curve during training on the Visdrone dataset. The blue curve represents Swin Transformer and the orange line represents FEA-Swin.

Our baseline model is Swin Transformer-tiny with FPN. The backbone network Swin-tiny has been pre-trained on Imagenet [47]. For both training and testing, we resized these images to a uniform size of 1000×600 with the keep_ratio set to true. We employed the AdamW optimizer and adjusted the weight decay to 0.05 and betas to (0.9, 0.999), respectively. We adopted the default 1x learning strategy in MMDetection, which iterates over 12 epochs. Considering that this is a medium-sized dataset, this is sufficient to train to convergence, and it also saves time and prevents overfitting. During the training phase, we set the initial learning rate to 0.001 and used a step learning strategy. Warmup is set to linearly scale at 0.001, causing the learning rate to increase at the beginning of training and reach a stable value in the middle. The learning rate starts to decrease at the eighth epoch and reaches 0.001 at the completion of the eleventh epoch. The loss curves during training on the Visdrone dataset are shown in Figure 9.

### 4.3. Results and Analysis

We used the average precision (AP) and mean average accuracy (mAP) as the evaluation to compare the accuracy of our method with other methods. AP is the average precision at 10 intersection over union (IoU) thresholds ranging from 0.5 to 0.95, with an equal division of 0.05 intervals. We counted ten categories of AP, and MAP is the average of all categories of AP. For all experimental results, we report the performance of the last epoch. It is specifically noted that our detection threshold of IoU is set at 0.6.

We first compare our method on two public datasets. Table 1 shows the results of FEA-Swin and the comparison models on the Visdrone dataset. The mAP of FEA-Swin is 7.3%, 6.9%, 10.5%, 14.4%, and 4.6% above that of Cascade R-CNN [7], Faster R-CNN [6], Retinanet [48], YOLOF [42], and Swin Transformer [21] on the Visdrone dataset, respectively. The results in the table show that all 10 categories gained significant AP improvement compared to these state-of-the-art methods on the Visdrone dataset.

**Figure 10:**
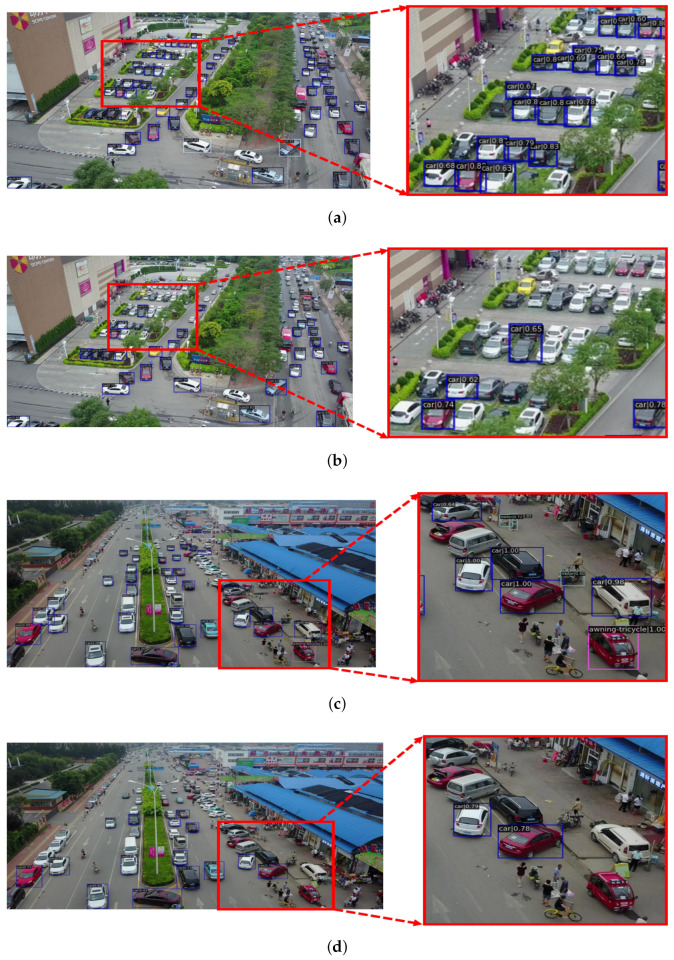
Selected comparison graphs of the detection results on the Visdrone dataset regarding Swin Transformer and FEA-Swin. The test maps for (**a**,**c**) used FEA-Swin. The test maps for (**b**,**d**) used Swin Transformer. On the left side of each column is the original detected picture, while on the right side is a zoom-in on the specific details on the left side.

Figure 10 shows a comparison of some selected detection results of the original Swin Transformer and FEA-Swin on the Visdrone dataset. From the diagram, it is clear that for densely packed objects in close proximity, our method has a substantial advantage. Furthermore, there were some adjacent dense targets with slight mutual occlusion or partial occlusion by complex backgrounds, particularly with regard to pedestrians and vehicles, which were also successfully detected.

**Figure 11:**
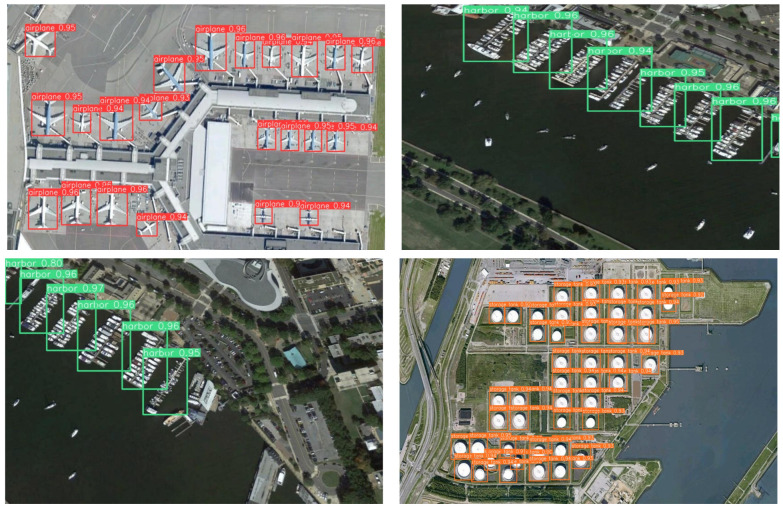
Some examples of detection results on the NWPU VHR-10 dataset using FEA-Swin.

Table 2 indicates that FEA-Swin achieves results over other state-of-the-art detectors on the NWPU VHR-10 dataset. It can be visualized from the table that the detection AP of FEA-Swin is 4.5%, 8.1%, 6.3%, 8.9%, and 2.4% higher than that of Cascade R-CNN [7], Faster R-CNN [6], Retinanet [48], YOLOF [42], and Swin Transformer [21] on the NWPU VHR-10 dataset, respectively. This indicator shows a massive improvement for aerial remote sensing image target detection. Figure 11 shows some detection results of FEA-Swin on the NWPU VHR-10 dataset. It can be seen that our method is very efficient in detecting small and dense objects (e.g., storage tanks). In addition, our detectors also show strong compatibility for rotating objects (e.g., airplanes).

**Figure 12:**
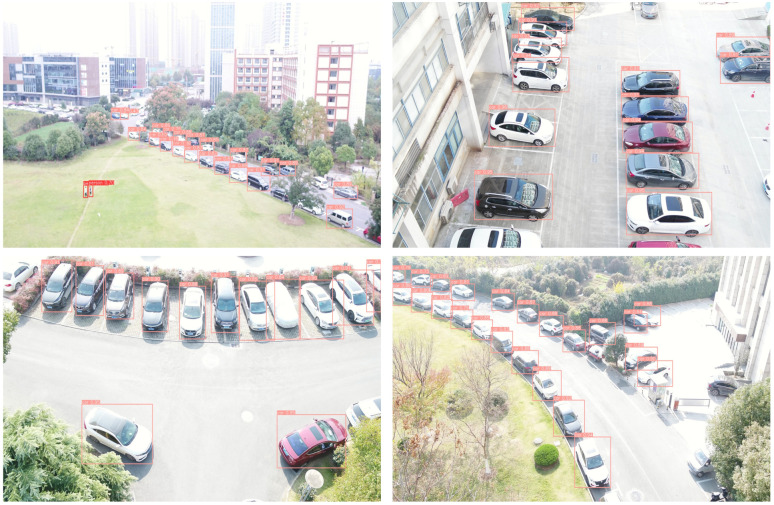
Some examples of detection results on our self-made dataset using FEA-Swin.

Similarly, Table 3 shows the favorable performance of FEA-Swin on our homemade dataset. Figure 12 shows some detection results of FEA-Swin on our self-collected dataset. It is evident that FEA-Swin can also have good detection performance on dense adjacent targets in simple real scenes containing pedestrians and vehicles. Even under intense light conditions, our method maintains a very satisfactory performance. Unfortunately, our FEA-Swin has a parameter amount of 121 M, which is 35 M more than the Swin Transformer’s 86 M. This means that our method increases the training and inference time marginally compared to the baseline method.

In general, FEA-Swin can precisely detect densely packed objects of different scales, including small objects (such as pedestrians and bicycles) and large objects (such as airplanes, ships, vans, and trucks) without geometrically increasing the number of model parameters.

### 4.4. Ablation Studies

**Figure 13:**
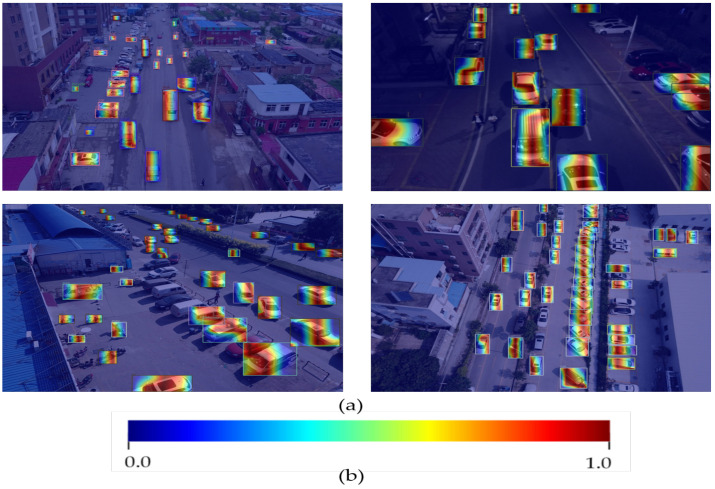
(**a**) A selection of class activation maps (with bounding boxes) exported using grad-cam. (**b**) A color tape corresponding to the model’s degree of focus. The redder the color, the more concern it holds; bluer the color, the less concern it holds.

In order to further understand the behavior of FEA-Swin, we implemented substantial ablation studies on the Visdrone dataset. We explored the influence of both BiFPN and FEAB components. The increments of mAP are list in Table 4. Adding our improved BiFPN component increases by 1.2% mAP in terms of detection accuracy compared with the baseline network, which indicates the importance of our improved BiFPN. It can be seen from Table 4 that the mAP significantly increases by 3.4% with the FEAB compared with the baseline network. We also can see that the AP improves significantly for each category, especially for dense objects, such as pedestrians and cars. Results in Table 4 show that the proposed components greatly improve the detection performance for objects with strongly similar characteristics, such as pedestrians and people, cars and vans, and tricycles and awning-tricycles. These results explicitly demonstrate the advantages of FEAB; it effectively improves the localization capability for dense object detection. To further demonstrate the ability of our FEA-Swin model to obtain feature information of aerial images, we used Grad-CAM [49] for the output of class activation maps, as shown in Figure 13. The class activation map shows where and how the weight or center of gravity shifts during the training of the model, and which part of the features the classification model is using to discriminate. In short, it mimics the process of human recognition of objects, finding the key parts of the relevant task as the model iterates. The darker the color in the picture, the more concern about the model. From the results, we conclude that FEAB makes a major contribution to improving the detection accuracy of dense objects.

### 4.5. Hyperparameter Independence of the Model

We discuss the effect of hyperparameters in this section to verify the parameter independence of the proposed method. To obtain controls for the different hyperparameter groups, we manually modified the hyperparameter values and recorded them to observe the effects on the self-collected dataset. We chose batch size, optimizer type, and weight decay coefficient as the three hyperparameters that are the most likely to affect the detector performance metrics. The consequences of our tuning are shown in Table 5. It can be derived that the detection effectiveness of FEA-Swin is not sensitive to definite changes in hyperparameters, which is a convincing validation of the hyperparameter independence of our method.

## 5. Conclusions and Future Work

This paper presents a novel transformer framework to accurately detect dense objects in UAV images. We designed a novel foreground enhancement attention Swin Transformer (FEA-Swin) framework to integrate context information to detect dense objects competently. We also improved a weighted bidirectional feature pyramid network (BiFPN) by designing a skip connection operation to keep abundant information about small objects. In addition, an efficient neck of the BiFPN network was introduced to balance the detection accuracy and efficiency by removing a redundant network layer.

Experiments show that the proposed object detection method can significantly improve the accuracy compared with state-of-the-art methods. Extensive ablation studies were conducted to further demonstrate the performance of the proposed method. In the future, we will explore both accurate and lightweight FEA-Swin in UAV object detection tasks.

## Figures and Tables

**Table 1:** Comparison results on VisDrone2021-DET-test-dev. The bolded numbers are the best indicators for each category.

Method	Backbone	Pedestrian	People	Bicycle	Car	Van	Truck	Tricycle	Awning- Tricycle	Bus	Motor	mAP
Cascade R-CNN	Resnet-50	16.8	9.1	11.8	59.2	31.4	32.7	12.9	15.6	58.1	16.7	26.4
Faster R-CNN	Resnet-50	16.7	9.1	10.4	59.3	34.1	33.0	17.2	15.3	57.6	15.1	26.8
RetinaNet	Resnet-50	15.5	9.1	9.8	58.9	28.7	27.9	7.5	5.0	55.6	13.8	23.2
YOLOF	Resnet-50	13.1	1.2	1.4	55.6	24.6	25.9	6.4	5.5	52.7	6.1	19.3
Swin Transformer	Swin-tiny	17.0	9.1	12.0	66.7	32.2	33.4	17.7	18.1	62.4	22.5	29.1
FEA-Swin	Ours	**31.1**	**14.6**	**13.5**	**70.1**	**42.4**	**39.3**	**19.1**	**18.5**	**62.7**	**25.6**	**33.7**

**Table 2:** Comparison results on NWPU VHR-10. The bolded numbers are the best indicators for each category.

Methods	Airplane	Ship	Storage Tank	Baseball Diamond	Tennis court	Basketball Court	Ground Track Field	Harbor	Bridge	Vehicle	mAP
Cascade R-CNN	95.3	90.4	**90.9**	**100**	90.6	89.3	98.1	99.7	61.1	71.9	88.7
Faster R-CNN	90.9	90.7	90.9	99.9	86.5	78.0	89.9	99.7	57.1	68.6	85.2
RetinaNet	95.6	64.3	76.2	94.9	82.3	51.3	97.7	82.6	56.7	67.2	76.9
YOLOF	98.3	87.9	89.2	97.8	82.6	74.6	98.5	88.9	60.7	64.8	84.3
Swin Transformer	**100**	88.0	90.8	**100**	90.3	88.4	**100**	99.7	78.8	71.9	90.8
FEA-Swin	**100**	**90.9**	**90.9**	99.9	**90.9**	**89.6**	**100**	**100**	**89.3**	**80.3**	**93.2**

**Table 3:** Comparison results on our self-collected dataset. The bolded numbers are the best indicators for each category.

AP	Method	Cascade RCNN	Faster RCNN	Retina Net	YOLOF	Swin Transformer	FEA-Swin
Class							
Car		84.1	81.6	78.4	79.6	88.5	**89.2**
Person		70.5	66.3	64.4	65.2	75.7	**79.5**
mAP		77.3	73.9	71.4	72.4	82.1	**84.4**

**Table 4:** Ablation studies of two key components of the Visdrone dataset. The bolded numbers are the best indicators for each category.

Settings	Pedestrian	People	Bicycle	Car	Van	Truck	Tricycle	Awning- Tricycle	Bus	Motor	mAP
Baseline	17.0	9.1	12.0	66.7	32.2	33.4	17.7	18.1	62.4	22.5	29.1
+BiFPN	28.2	9.3	12.2	68.2	41.7	**40.7**	18.5	17.2	48.5	18.3	30.3
+FEAB	30.9	12.6	12.9	**70.2**	42.2	38.7	18.7	**18.7**	52.7	**27.5**	32.5
+BiFPN+FEAB	**31.1**	**14.6**	**13.5**	70.1	**42.4**	39.3	**19.1**	18.5	**62.7**	25.6	**33.7**

**Table 5:** Hyperparametric independence study for FEA-Swin on our self-collected dataset.

Method	Batch Size	Optimizer Type	Weight Decay	mAP
FEA-Swin-v1	4	SGD	0.05	83.6
FEA-Swin-v2	4	AdamW	0.05	84.4
FEA-Swin-v3	4	AdamW	0.1	84.3
FEA-Swin-v4	8	SGD	0.05	83.4
FEA-Swin-v5	8	AdamW	0.05	84.1
FEA-Swin-v6	8	AdamW	0.1	83.9

## Data Availability

The Visdrone and NWPU VHR-10 datasets used in this paper are public datasets. The link to download our home-made dataset is https://pan.baidu.com/s/14UcfTtZnvvVyCV2tAzHFKw (accessed on 11 August 2022), the verification code is wjy8.
